# Renal Artery Perforation During Transcatheter Aortic Valve Replacement

**DOI:** 10.1016/j.jaccas.2025.105299

**Published:** 2025-10-08

**Authors:** Saleh Altaf, Hammad Shafique, Alexander Tindale, Konstantinou Konstantinos, Tito Kabir

**Affiliations:** Cardiology Department, Harefield Hospital, Guy's and St Thomas' NHS Foundation Trust, London, United Kingdom

**Keywords:** hemorrhagic shock, renal artery perforation, TAVR complication

## Abstract

**Background:**

Transcatheter aortic valve replacement (TAVR) is a well-established modality to treat severe aortic stenosis.

**Case Summary:**

We report a case of a woman in her 70s who underwent TAVR. The procedure was complicated owing to right renal artery perforation by a 0.038-inch angled-tip hydrophilic guidewire during closure of the left femoral artery, requiring implantation of a covered stent in renal artery to achieve hemostasis.

**Discussion:**

In cases of shock during TAVR, injury to nonaccess site vessels should be considered. Treatment of iatrogenic renal artery injury with covered stent deployment achieves rapid hemostasis and preserves kidney function.

**Take-Home Messages:**

In case of periprocedural hemorrhagic shock during TAVR, injury to nonaccess site vessels should be considered. Meticulous fluoroscopic guidance should always be used when advancing guidewires to prevent vascular damage. Awareness of patient-specific anatomical variation of arteries can help the operator to be more cautious and avoid entering that artery.

**Visual Summary:**
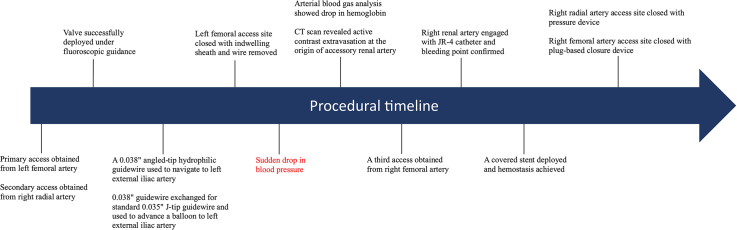
Procedural Timeline CT = computed tomography.

## History of Presentation

A woman in her late 70s was referred to the cardiology clinic for several months of progressively worsening dyspnea on exertion. Her past medical history was remarkable only for hypertension and shellfish allergy. On physical examination, a loud ejection systolic murmur was audible in the second intercostal space at the right sternal border, with radiation to the carotid arteries. Her echocardiography confirmed severe aortic stenosis with preserved left ventricular function. A diagnostic coronary angiogram demonstrated a severe lesion in ostial left main coronary artery. Therefore, 1 month before a transcatheter aortic valve replacement (TAVR) procedure, the patient had a balloon aortic valvuloplasty and percutaneous coronary intervention to the left main coronary artery.

**Figure 1:**
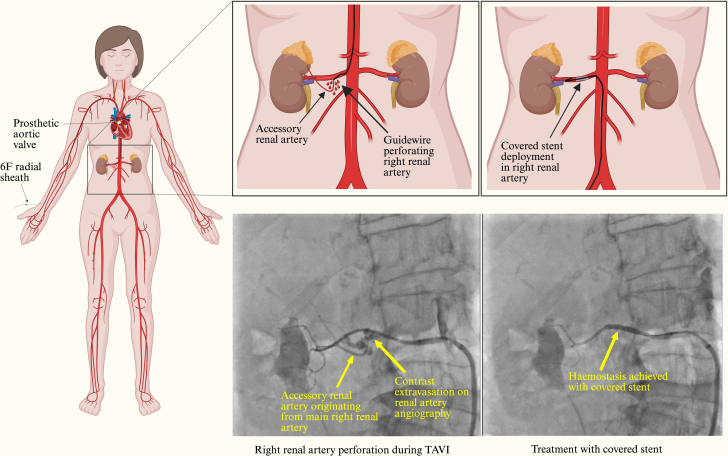
Mechanism of Injury and Management With Covered Stent TAVI = transcatheter aortic valve implantation.

The patient then attended for her TAVR procedure. After primary access was obtained through the left femoral artery and secondary access was obtained through right radial artery, a Sapien S Ultra 26-mm valve (Edwards Lifesciences) was deployed under rapid pacing from a stiff guidewire in the left ventricle. A dry closure technique was employed to close the left femoral artery access site, which involved advancing a 0.038-inch angled-tip hydrophilic guidewire (Radifocus, Terumo UK) from the right radial artery down to the left external iliac artery under fluoroscopic guidance. The guidewire briefly entered some aortic side branches before being repositioned to the left external iliac artery; however, no resistance or unusual features were noted during repositioning. This was exchanged for a 0.035-inch standard J-tip guidewire via a 6-F pigtail catheter. A 12-mm Armada balloon (Abbott Medical UK) was then advanced over the 0.035-inch standard J-tip guidewire and was inflated in the left external iliac artery until pressure reduction to <40 mm Hg was achieved on the femoral arterial tracing. After closure of the left femoral access site with 2 Perclose ProStyles (Abbott Medical UK) and an Angio-Seal (Terumo UK) over a standard J-wire used to protect the primary access site, the patient developed acute hypotension, with blood pressure of 60/40 mm Hg (Figure 1).

## Differential Diagnosis and Investigations

**Figure 2:**
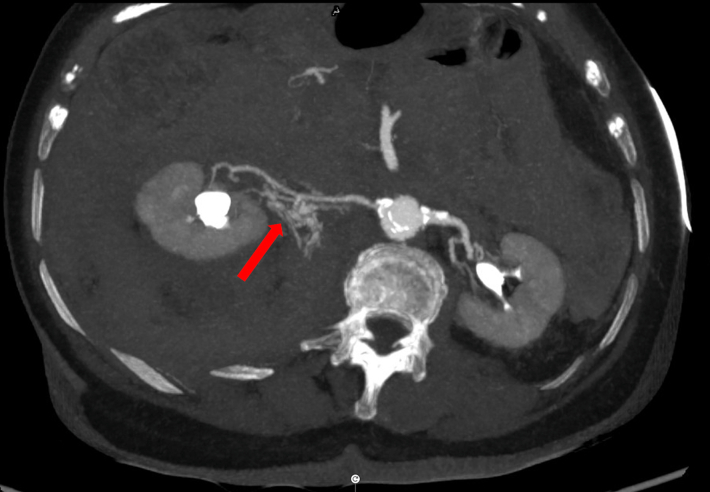
Cross-Sectional Computed Tomography Image of the Abdomen Showing Contrast Extravasation From the Right Renal Artery (Red Arrow)

An aortogram showed an optimally positioned valve with no transvalvular or paravalvular leak. No aortic annular rupture or obvious aortic dissection flap was identified. Transthoracic echocardiography confirmed a well-seated prosthetic valve with no regurgitation, a hyperdynamic left ventricle with only a trivial pericardial effusion, and a collapsing inferior vena cava. A digital subtraction angiogram from the aortic root to the left femoral artery did not show any vascular injury or bleeding point. Coronary angiography demonstrated some haziness in the left main coronary artery, suggesting a possible thrombus. The remainder of the coronary arteries were unobstructed. Given the paucity of obvious causes of such profound hypotension, a drug-eluting stent was deployed in the left main coronary artery, but this intervention did not improve the blood pressure. The patient was treated with 3 boluses of 25 μg of intravenous adrenaline and 100 mg of intravenous hydrocortisone to treat possible anaphylactic shock from intraprocedural medications (cefuroxime, teicoplanin, and protamine). The patient's blood pressure temporarily improved but dropped again. Subsequently, a continuous infusion of adrenaline was used for vasopressor support. Arterial blood gas analysis revealed hemoglobin of 4.8 g/dL and lactate of 1.5 mmol/L. Compared with preprocedure hemoglobin of 11.3 g/dL, it was a significant drop. To identify the source of bleeding, a whole-body computed tomography (CT) scan with contrast was performed, which identified active contrast extravasation at the origin of an accessory renal artery from the main right renal artery (Figure 2). There was no abnormal dilatation, aneurysm, or dissection of renal arteries. Perfusion of both kidneys was normal.

## Management

**Figure 3:**
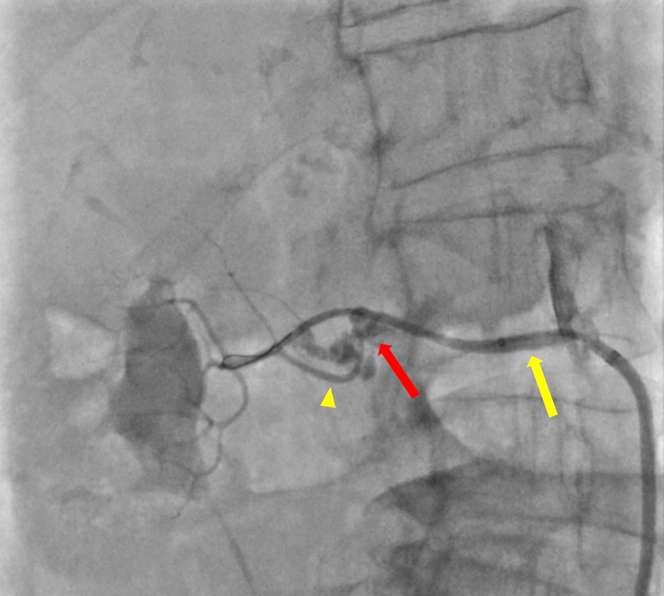
Angiography of the Right Renal Artery Showing Contrast Extravasation (Red Arrow) at the Origin of the Accessory Renal Artery An accessory renal artery can be seen (yellow arrowhead) originating from the right renal artery (yellow arrow).

**Figure 4:**
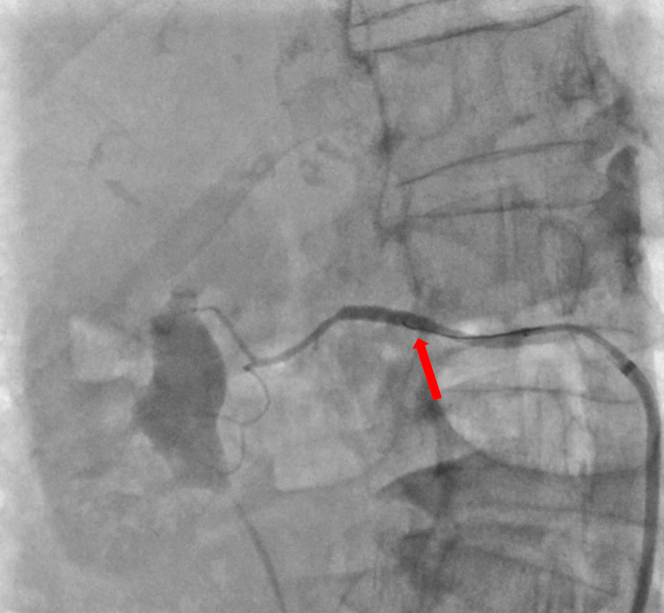
Angiography Showing Adequate Hemostasis With Covered Stent Deployed in the Right Renal Artery (Red Arrow) Absence of accessory renal artery, visible in Figure 3, can be appreciated.

Two units of packed red blood cells were rapidly transfused, and emergency transfer to the theater for vascular surgery was arranged. However, even in this short time window the hemodynamic situation worsened, with unrecordable blood pressure and reduced consciousness, therefore the patient was taken back to the catheterization laboratory. A third access site was obtained from the right femoral artery, and the bleeding point at the origin of the right accessory renal artery (a branch of the main right renal artery) was identified on angiography (Figure 3). The right renal artery was engaged with a Judkins right 4.0 catheter, expediently wired using an Asahi Sion blue guidewire (Asahi Intecc), and a 3.5 × 20 mm PK Papyrus covered stent (Biotronik UK) was deployed. After this, hemostasis was confirmed on fluoroscopy (Figure 4). The right femoral arterial access site was closed with an Angio-Seal, and the right radial access site was closed using a pressure band. The patient was transferred to intensive care unit for further care.

## Outcome

Prior to discharge, ultrasound of the kidneys showed normal shape and size of both kidneys, with well-maintained corticomedullary differentiation. Transthoracic echocardiography revealed a well-seated prosthetic valve. The patient's hemoglobin was 9.1 g/dL (baseline: 11.3 g/dL), and serum creatinine remained normal throughout admission. At the 3-month follow-up, she was clinically well, with an unremarkable echocardiogram and renal ultrasound.

## Discussion

The aim of this case report is to highlight an unusual vascular injury that occurred in our patient during TAVR. Vascular injuries are among the most common complications of TAVR. Data from large registries and meta-analyses indicate that the risk of vascular injury is 0.94% to 11.9%, with access site complications being the most common,^1^^,^^2^ but there is a paucity of data on the frequency of injury to individual nonaccess site arteries and subsequent management strategies to prevent damage to the organ supplied.

To the best of our knowledge, there is only 1 other case report of renal artery perforation during TAVR, by Kilic et al.^3^ The investigators postulated that perforation of the renal artery occurred from the 0.035-inch J-tip guidewire during postclosure angiography of the femoral artery access site, and deviation of abdominal aorta to the right side was identified as the risk factor, making manipulation of the guidewire challenging. In comparison, renal artery injury in our patient occurred from a 0.038-inch angled-tip hydrophilic guidewire during dry closure of the femoral access site. CT scan and renal angiography identified an accessory renal artery originating from the main right renal artery, with bleeding at the point of origin accessory renal artery. We hypothesize that this anatomical variant, present in 8.34% of the general population,^4^ was a contributing factor leading to perforation.

To avoid a similar complication, the following 3 precautions can be taken:

1. Use of safer guidewires: Operators should aim to use a standard J-tip guidewire to cross into the descending aorta from the right subclavian artery, rather than use a hydrophilic guidewire first-line. A standard J-tip guidewire with large primary curve is safer, with less risk of inadvertently entering side branches, thereby reducing the risk of injury. The hydrophilic guidewires should only be used once attempts with a standard guidewire have been exhausted.
2. Meticulous fluoroscopic guidance: The tip of the guidewire should always be visible on the fluoroscopy screen while advancing the guidewire, to avoid entering deep into the side branches.
3. Awareness of patient-specific arterial anatomy: The presence of any arterial anatomical variation, found incidentally on CT angiogram for TAVR planning, should make operators more cognizant of patient-specific arterial anatomy, and caution should be taken to avoid entering that artery.

Management of iatrogenic renal artery injury, primarily discussed in the interventional radiology literature, can be summarized as follows:

1. Balloon tamponade: Inflating a balloon in the renal artery can provide temporary hemostasis and help to hemodynamically stabilize the patient until more expert help from interventional radiologists and vascular surgeons is available. Balloon tamponade alone has been shown to achieve permanent hemostasis in a case series.^5^
2. Covered stent deployment: If the proximal portion of the renal artery with relatively bigger caliber is perforated, a covered stent placement can be used to seal the perforation.^6^ This strategy was used in our patient. It preserves blood flow to the renal parenchyma but can be difficult in distal small caliber branches.
3. Arterial embolization: In case of perforation in a distal small caliber vessel, arterial embolization can be used to secure hemostasis.^7^
4. Surgery: Surgical intervention might be needed, especially in hospitals where expertise for less invasive procedures is not available.^8^

## Conclusions

Our case demonstrates a rare vascular complication of TAVR. It highlights the risk of injury to nonaccess site vessels, especially if there is a variation in arterial anatomy, and emphasizes the importance of meticulous fluoroscopic guidance.

**Take-Home Messages**

• In case of peri-procedural hemorrhagic shock during TAVR, injury to non-access site vessels should be considered.
• Meticulous fluoroscopic guidance should always be used when advancing guidewires to prevent vascular damage.
• Awareness of patient-specific anatomical variation of arteries can help operator to be more cautious and avoid entering that artery.

## Funding Support and Author Disclosures

The authors have reported that they have no relationships relevant to the contents of this paper to disclose.
